# Rapid brain lymphoma diagnostics through nanopore sequencing of cytology-negative cerebrospinal fluid

**DOI:** 10.1007/s00401-024-02793-z

**Published:** 2024-09-03

**Authors:** J. Hench, C. Hultschig, I. Bratic Hench, H. Sadasivan, Ö Yaldizli, G. Hutter, S. Dirnhofer, A. Tzankov, S. Frank

**Affiliations:** 1 Institute for Pathology and Medical Genetics, University Hospital Basel, Basel, Switzerland; 2 AI Group, AMD and Paul G Allen School of CSE, University of Washington, Seattle, USA; 3 Dept. of Neurology, University Hospital Basel, Basel, Switzerland; 4 Dept. of Neurosurgery, University Hospital Basel, Basel, Switzerland

**Keywords:** Liquid biopsy, CNS lymphoma, Rapid testing, Nanopore sequencing, DNA methylation, Tumor classification, Non-invasive diagnostics, Unsupervised machine learning, Epigenetics, Point-of-care testing

Haematological neoplasms affecting the CNS, most prominently aggressive B-cell lymphomas, require rapid diagnosis, typically by stereotactic biopsy, to initiate treatment, prompting non-invasive modalities [6]. In challenging cases, DNA methylation (DNAmeth) and copy number variant (CNV) profiling of cerebrospinal fluid (CSF) may meet this demand. Healthy and neoplastic cells, including CNS lymphomas [11], may shed DNA fragments into the bloodstream and CSF as cell-free DNA (cfDNA). In addition, cellular debris can contribute to sediment DNA (seDNA). In several paediatric high-grade brain tumours, CSF contains sufficient amounts of tumour-derived cfDNA (cf-tDNA) for methylation- and CNV-based tumour classification through ligation-based nanopore sequencing [1], and for disease monitoring by various approaches [15]. Currently, these methods require laborious sample processing and expensive infrastructure.

Here, we have CSF-adapted our fast-track unsupervised machine learning (ML) approach [9] for cases with a differential diagnosis of lymphoma and other malignant brain tumours including metastases. We demonstrate its clinical application in two CNS-lymphoma cases. Comparison of nanopore sequencing-derived methylation patterns to pan-cancer epigenomic and CNV data allowed next-day diagnosis [9] and treatment initiation. Our point-of-care protocol can significantly reduce neurological impairment through timely and non-invasive testing. In short, we have adapted CSF preservation (Cell-Free DNA BCT CE, Streck, USA) and cfDNA extraction protocols (MagMAX™, Cat: A29319; KingFisher™ Cat: 5400110, Thermo Fisher, USA) to comply with a wide range of clinical scenarios (Fig. 1a); CSF seDNA was extracted as described (DNeasy kit ID: 69504; QiaCube, Qiagen, Germany) [9]. Sequencers were controlled, SQK-RBK004 kit (ONT, UK) was used, and data were analysed with NanoDiP as previously described [9] (Fig. 1a, open source code: see material availability). Patient characteristics are summarised in Table 1.

**Fig. 1:**
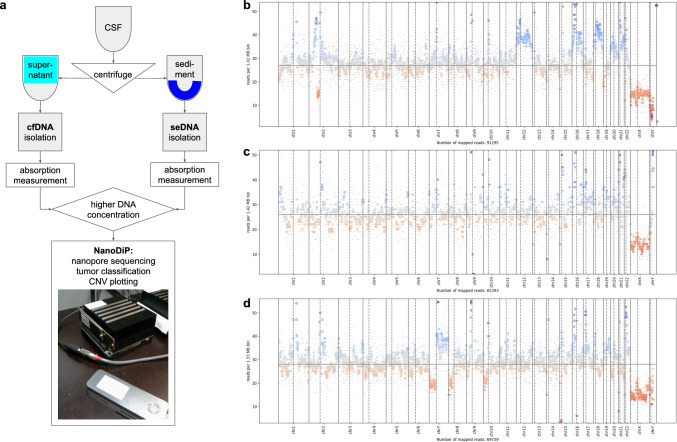
CSF analysis workflow and resulting CNV plots. **a** CSF drawn into a cfDNA preservation tube is centrifuged to separate sediment from supernatant. cfDNA and seDNA are extracted separately using commercial systems for cfDNA (supernatant) or tissue/cells (sediment). Photometric absorption measurement quantifies DNA. The fraction with the higher amount of DNA is subjected to NanoDiP analysis. Photograph demonstrates the small footprint of the NanoDiP device, consisting of a Jetson (Nvidia, USA) AGX Xavier computer (black box, top) to which an Mk1B (silver box, bottom) sequencer (ONT, UK) is connected through a USB3 cable. Sequencer control and all downstream data analysis occurs offline on the Jetson computer. Sequencing and reference methylation data are contained on the solid state drive attached to the Jetson computer (PCIe module on the left edge of the black box). **b**, **c** CNV plots, case 1. a: cfDNA, b: seDNA. Note the amplitude difference in both CNV profiles relative to the chromosome X vs. baseline difference. While cfDNA mainly consists of cf-tDNA, as confirmed independently by ddPCR, the seDNA does not contain significant amounts of tumour DNA. The seDNA likely stems from admixed leukocytes. Respective methylation profile had similarities to reactive lesions (not shown). **d** CNV plot from seDNA of case 2. Note the copy number alterations that indicate a clonal, neoplastic cell population. The amplitude of the aberrations is high relative to the chromosome X vs. baseline amplitude, suggesting a high contribution of neoplastic cell DNA to the seDNA

**Table 1:** Case summary

Case	Sex	Age [y]	Clin. differential	cfDNA [ng/µl]	seDNA [ng/µl]	Fraction analysed	Run [h]	Integrated diagnosis	Ancillary findings	Cytology
1	m	68	Multifocal CNS lymphoma or glioma	~ 10	~ 4	cfDNA	17.5	DLBCL	MYD88: p.L265P, AF 95%	Negative
2	m	48	NK/TCL recurrence in CNS	~ 4	~ 9	seDNA	16.5	NK/TCL	Clonal TCR rearr.	Sparse unclassifiable lymphoid cells, FACS inconclusive
3	f	8	AT/RT recurrence	~ 4	n.d	cfDNA	17.6	AT/RT (recurrent)	-	2 × negative
4	m	71	Unclear intracerebral mass lesion; lymphoma, glioma, other?	~ 3	< 1	cfDNA	19.5	Inconclusive	Biopsy: glioblastoma, IDHwt	n.d
5	w	72	Lymphoma vs. glioma	~ 2	~ 1	cfDNA	14.8	Inconclusive	Biopsy: glioblastoma, IDHwt	n.d
6	m	67	Glioblastoma IDHwt 1.5y prior; recurrence vs. radionecrosis?	~ 6	~ 2	cfDNA	42.8	Inconclusive	Biopsy: glioblastoma, IDHwt	n.d

## Case 1

Biopsy of a small periventricular lesion, suspicious for CNS lymphoma, revealed reactive changes. Following negative CSF cytology, a second CSF spinal tap sample submitted as a “liquid biopsy” revealed elevated cfDNA in the supernatant. CNV (Fig. 1b) and DNAmeth profiles (100% match) of cfDNA completed the next day suggested diffuse large B-cell lymphoma, enabling initiation of immediate treatment. Droplet Digital PCR (ddPCR) for *MYD88*:p.L265P [6, 10] (Cat# WT:10042967/ MUT:10042964, BioRad, USA, manufacturer-supplied protocol) was positive (same cfDNA extract and day), independently supporting the diagnosis. Post hoc nanopore sequencing and ddPCR of seDNA (research purpose) showed a flat CNV, unspecific DNAmeth profiles (Fig. 1c), and negative *MYD88*-ddPCR.

## Case 2

Suspected leptomeningeal relapse of an extranodal EBV-positive NK/T-cell lymphoma (NK/TCL) diagnosed 10 months ago. CSF cytology was negative. A subsequent CSF spinal tap “liquid biopsy” had higher seDNA than cfDNA; seDNA was sequenced. Due to a lack of T-cell lymphoma references in our pan-cancer database, the DNAmeth profile remained unclassifiable, whilst the CNV plot revealed alterations recurrently found in NK/TCL [12], warranting the integrated diagnosis of NK/TCL relapse (Fig. 1d). Blood analysis by a parallel sequencing panel revealed clonal T-cell receptor gamma rearrangement 9 days later (Cat: A51562, Thermo Fisher, USA).

## Case 3

Child with atypical teratoid/rhabdoid tumour (AT/RT; CNS WHO grade 4) diagnosed 5.5 months ago. CSF monitoring cytology was negative twice. A third “liquid biopsy” CSF spinal tap displayed elevated cfDNA and nanopore sequencing revealed the DNAmeth profile of ATRT_TYR [4] as well as CNVs already established from the primary tumour tissue specimen. This case demonstrates the general applicability of our diagnostic CSF analysis workflow.

## Cases 4, 5, 6

Adult patients with molecularly confirmed glioblastoma, *IDH* wildtype (GB-*IDH*wt), with radiological and clinical differential diagnoses including lymphoma in cases 4 and 5 (Table 1). CSF spinal tap cfDNA sequencing remained diagnostically inconclusive in each case. These patients were included to illustrate the limitation of our CSF analysis workflow, most likely due to insufficient cfDNA fractions released from GB-*IDH*wt cells.

Discrimination of CNS lymphoma from other neuroradiological mimics including small-cell cancers and non-neoplastic conditions such as encephalitis is of utmost clinical importance. At least to some extent, our approach can detect inflammatory signatures [9] and pinpoint neoplasia based on CNVs. Moreover, clearance of cf-tDNA from CSF may reflect a sustained tumour response in CNS-lymphoma patients [8], making our approach a potential monitoring tool, even though it is mainly qualitative.

Its universal applicability, low hands-on, and infrastructure requirements [9] may help to reduce the number of patients receiving steroids before lymphoma diagnosis. Such pretreatment often massively delays or even prevents timely CNS-lymphoma diagnosis. In cases with two positive confirmatory results (nanopore sequencing/ ddPCR), CNS biopsy may even be omitted bearing in mind that surgical complication rates of stereotactic brain biopsies of ~ 5%, mainly haemorrhages, negatively impact outcomes [3].

The sensitivity of our workflow under concurrent corticosteroid therapy remains to be elucidated. In urgent clinical settings, steroid therapy could theoretically be initiated as soon as an elevated CSF cfDNA content, suitable for further analysis, has been established. Importantly, CSF sampling and shipment at ambient temperature to a laboratory for sequencing can be completed within 24 h.

Whilst the small cohort size is a clear limitation, our observations still highlight the utility of nanopore CSF workup and call for democratising accessibility of high-quality DNAmeth/CNV-reference datasets to broaden applicability. Given the low rates at which CNS-lymphoma diagnoses can be obtained through conventional CSF analyses [2, 14], our findings could prove transformative for the clinical management of patients with suspected CNS lymphoma.

## Data Availability

Due to changes in nanopore sequencing chemistry, we adapted methylation calling algorithms [9] to the current “RBK-114” rapid sequencing kit on MinION and PromethION (Oxford Nanopore Technologies, UK). Analysis tools were adapted to the ORIN Developer Kit (Nvidia, USA) [5, 7, 13]. **Source: **https://github.com/neuropathbasel/nanodip_dev

## Data availability

Source code is available at: https://github.com/neuropathbasel/nanodip_dev
